# Tailoring thermal insulation architectures from additive manufacturing

**DOI:** 10.1038/s41467-022-32027-3

**Published:** 2022-07-25

**Authors:** Lu An, Zipeng Guo, Zheng Li, Yu Fu, Yong Hu, Yulong Huang, Fei Yao, Chi Zhou, Shenqiang Ren

**Affiliations:** 1 Department of Mechanical and Aerospace Engineering, University at Buffalo, The State University of New York, Buffalo, NY USA; 2 Department of Industrial and Systems Engineering, University at Buffalo, The State University of New York, Buffalo, NY USA; 3 Department of Materials Design and Innovation, University at Buffalo, The State University of New York, Buffalo, NY USA; 4 Department of Chemistry, University at Buffalo, The State University of New York, Buffalo, NY USA; 5 Research and Education in Energy, Environment & Water (RENEW) Institute, University at Buffalo, The State University of New York, Buffalo, NY USA

**Keywords:** Synthesis and processing, Polymers, Organic molecules in materials science

## Abstract

Tailoring thermal transport by structural parameters could result in mechanically fragile and brittle networks. An indispensable goal is to design hierarchical architecture materials that combine thermal and mechanical properties in a continuous and cohesive network. A promising strategy to create such a hierarchical network targets additive manufacturing of hybrid porous voxels at nanoscale. Here we describe the convergence of agile additive manufacturing of porous hybrid voxels to tailor hierarchically and mechanically tunable objects. In one strategy, the uniformly distributed porous silica voxels, which form the basis for the control of thermal transport, are non-covalently interfaced with polymeric networks, yielding hierarchic super-elastic architectures with thermal insulation properties. Another additive strategy for achieving mechanical strength involves the versatile orthogonal surface hybridization of porous silica voxels retains its low thermal conductivity of 19.1 mW m^−1 ^K^−1^, flexible compressive recovery strain (85%), and tailored mechanical strength from 71.6 kPa to 1.5 MPa. The printed lightweight high-fidelity objects promise thermal aging mitigation for lithium-ion batteries, providing a thermal management pathway using 3D printed silica objects.

## Abstract (web summary)

Nanoscale porous building blocks hold great promise for a range of thermal management technologies but often porous nanoparticle assemblies are intrinsically brittle. Here, the authors report 3D printing of porous silica voxels with polymeric additives to enable the coupling between additive manufacturing and hierarchical assembly for superior machinability and structural controllability.

## Introduction

Additive manufacturing prototypes highly customized objects with complex geometry and programmable structures^1–4^, while the bottom-up control from modular building blocks enables additively manufactured architectures exhibiting technically important functionalities^1,2,5,6^. Nanoscale porous building blocks, the outstanding attributes of large surface areas, high porosity, and low density, regulate thermal transport in the architectured structures within the mean free path of air particles (Knudsen effect), holding great promise for a range of thermal management technologies. However, porous nanoparticle assemblies are intrinsically brittle because of its weak interparticle interactions, limiting its structural shaping, machinability and high-fidelity miniaturization^1,7^. Therefore, tailoring hierarchical assembly structure from nanoscale porous building blocks is critical to meet the desired mechanical and thermal properties, high-fidelity and multi-scalability of highly miniaturized structures. Additive manufacturing of hybrid material assembly, non-covalent bridging inorganic and organic units, is a promising strategy to engineer mechanical robustness and thermal insulation of porous nanoparticle assembly architectures in advanced thermal management systems.

**Fig. 1:**
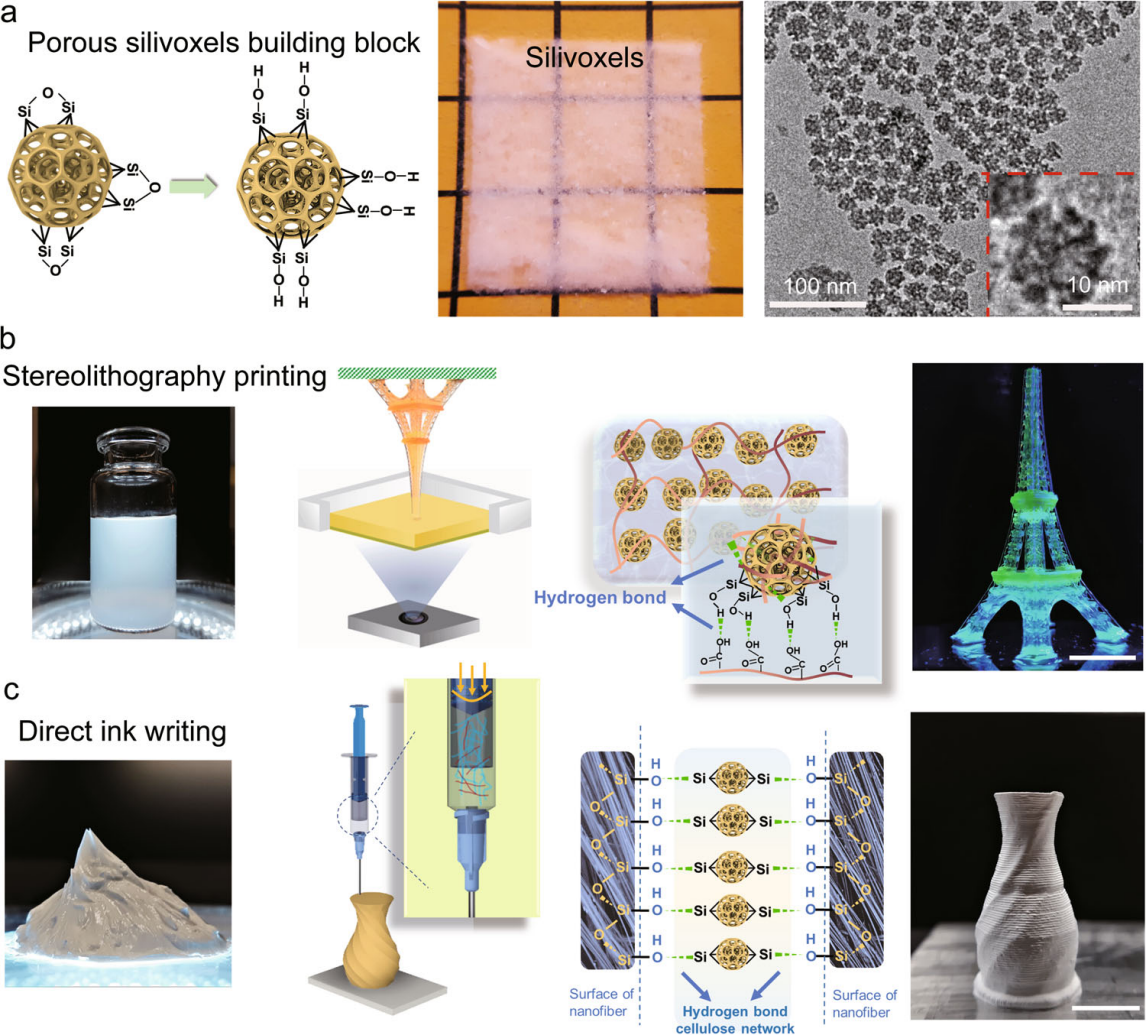
Porous silivoxels for additive manufacturing of thermal insulation materials. **a** The porous silivoxel powders as the feedstock, and the slurry of polymer additives and porous silivoxels as the ink for additive manufacturing. The corresponding TEM image of porous silivoxels. **b** Mechanically tunable silivoxels through tuning the hydrogen-bonding polymer chains and printed via stereolithography process. The mechanical flexible miniaturized Eiffel tower composed of silivoxels with high resolution. **c** Mechanical robust miniaturized vase with high shape fidelity via direct ink writing. Incorporating fiber can reinforce the strength of DIW-silivoxels. Scale bars represent 10 mm.

Here we report the versatile orthogonal surface hybridization of porous silica voxels (silivoxels) with polymeric additives to enable the coupling between additive manufacturing and hierarchical assembly for superior machinability and structural controllability. The uniform porous silivoxels are synthesized via the hydrolysis and condensation of tetraethyl orthosilicate (TEOS) on cetyltrimethylammonium bromide (CTAB) micelles swollen with mesitylene, where mesitylene acts as the pore expander of the CTAB micelles^8^. Figure 1a shows transmission electron microscopy (TEM) images of porous silivoxels, while the well-defined porous structures can be maintained after high-temperature sintering treatment (Supplementary Figs. 1 and 2). The specific surface area (SSA) and pore sizes of porous silivoxels are characterized via the Brunauer–Emmett–Teller (BET) technique (Supplementary Fig. 3). Through changing the reaction temperature from 0 °C to 100 °C and post-sintering from 480 °C to 600 °C, the pore size of silica ranges from 8 nm to 11 nm while the SSA ranges from 516 m^2^/g to 210 m^2^/g (Supplementary Fig. 3). Due to the synergistic contribution of small pore sizes and large SSA (~516 m^2^/g), porous silivoxels reach a thermal conductivity of 19.1 mW m^−1^ K^−1^. Porous and functionalized silivoxels are mechanically modulated through the penetration of polymeric additives to produce a 3D hierarchical hybrid network, where the collective interactions of silica hybrid voxel networks could maintain the integrity of silivoxel assemblies. Such mechanically tailored and printed insulation architectures generate a compelling opportunity to build resilient thermal mitigation systems under extreme conditions. The convergence of agile additive manufacturing and porous silivoxels provides a holistic solution to address the aforementioned challenges to tailor mechanical robustness without sacrificing high thermal insulation performance.

## Result

The hierarchical porous nature in silivoxels is featured by high surface area and open-pore structures, opening up the pathway for surface and open-pore polymer chain hybridization to form a percolated silica network reinforced by interfacial hydrogen bonding interactions. Figure 1 illustrates hydrogen-bonding hybridization of porous silivoxels with the hydroxyl end groups of poly(ethylene glycol) diacrylate (PEGDA) and hydroxypropyl methyl cellulose (HPMC)^9,10^ formulating the tailorable inks for the direct assembly of hierarchical porous objects by means of stereolithography (SLA-Silivoxel, Fig. 1b) or direct ink writing (DIW-Silivoxel, Fig. 1c), respectively. The agile additive manufacturing of silica objects enables a tunable mechanical performance as a result from the hydrogen bonding between silanol groups of silivoxel surface and the ether oxygens or hydroxyl end groups in polymer additives such as PEGDA or cellulose^9^. The mechanical robustness is obtained through the enhanced interparticle interactions between silivoxels via the polymer additives, while the mechanical flexibility is achieved through the hybrid network coupled by the physical interchain hydrogen bonds and chemically covalent cross-linking. On the other hand, the reduced volume of the solid phase in porous silica can contribute to the reduction in the heat conduction pathways, thus providing the printed objects with low thermal conductivity^11,12^.

**Fig. 2:**
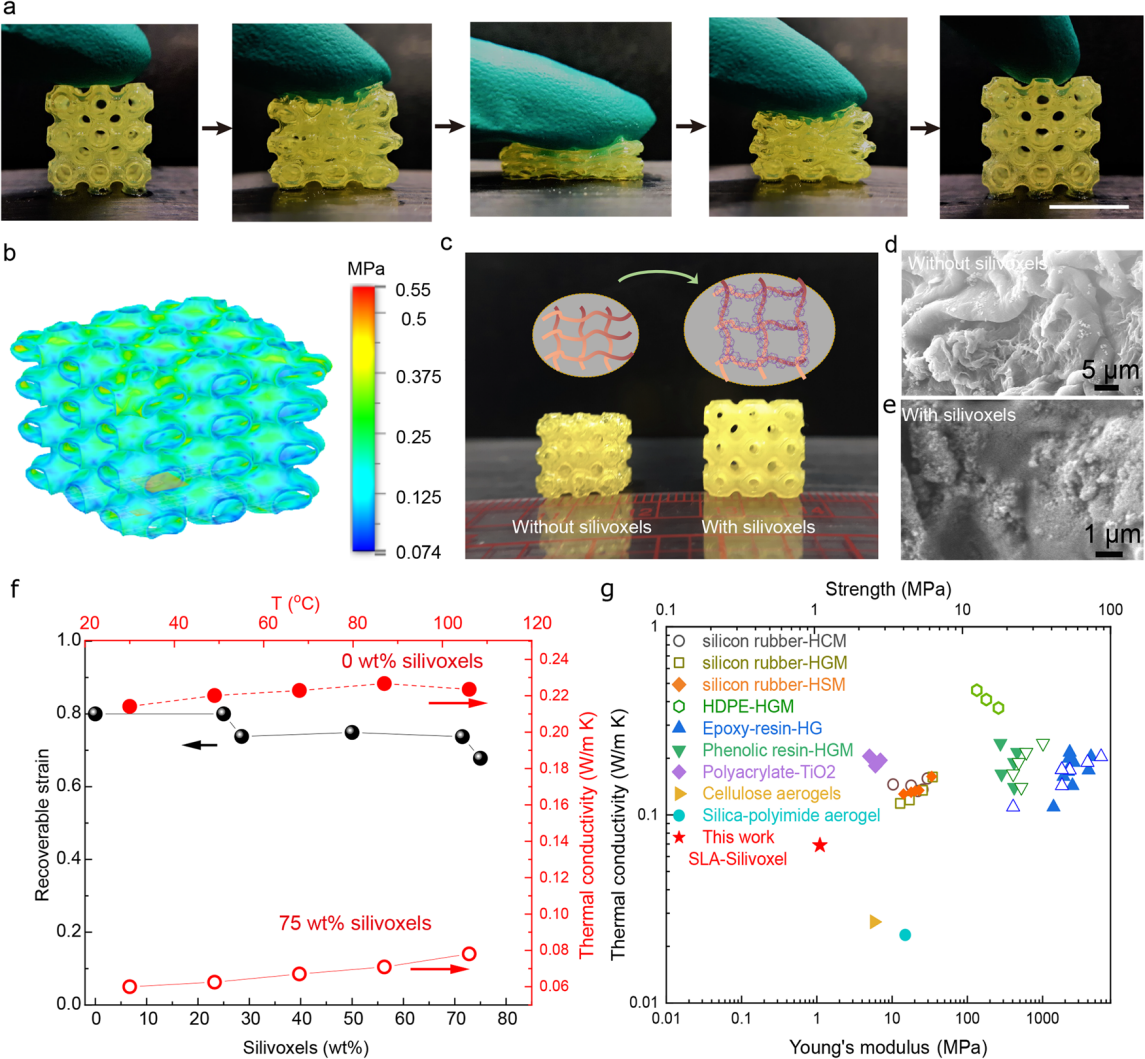
Characteristics of SLA-silivoxels. **a** The compressive recoverable SLA-silivoxels lattice with 70 wt% silivoxel concentration. Scale bar is 10 mm. **b** FEA (Finite element analysis) stress contour of uniaxial compression of SLA-silivoxels. **c** The optical images of dried SLA samples with and without silivoxels. The SLA-silivoxels can maintain the shape while SLA samples without silivoxels shrink significantly. **d** SEM image of SLA sample without silivoxels. **e** SEM image of SLA-silivoxels. **f** Compression recoverable strain vs. silivoxels concentration, and temperature-dependent thermal conductivity of SLA samples with 0 wt% and 75 wt% silivoxels. **g** Comparison of thermal conductivity vs. Young’s modulus (solid symbols) and Strength (open symbols) between literature and SLA-silivoxels in this work.

The translucent and flexible hybrid silica objects are printed at a rapid speed (4 mm/min) and high spatial resolutions (50 µm) through the mask image projection based stereolithographic 3D printing (SLA-Silivoxles), including the pyramid, the gyroid structure, and the miniaturized Eiffel tower (Fig. 1b and Supplementary Fig. 6). The crosslinked PEGDA network enables mechanical toughness of SLA-Silivoxels, while interfacial bonding between silica and PEGDA network dissipates energy upon deforming SLA-Silivoxels. The presence of silivoxels not only increases the cross-linking density by interacting with the PEGDA chains, but also renders its superior thermal insulation properties. Figure 2a is finger compression/release of the triply periodic minimal surfaces (TPMS) structure (the Schwarz primitive model) with zero mean curvature and smooth minimal surface transition, demonstrating the flexibility of SLA-Silivoxels^13,14^. The compression flexibility is resulted from the synergistic effect of hydrogen-bonded silica voxel assembly and the crosslinked polymeric network in SLA-Silivoxels. Due to the reversible non-covalent interactions (hydrogen bonds, chain entanglements, and van der Waals interactions) between silivoxels and PEGDA chains, mechanical stress on SLA-Silivoxels could be effectively dissipated by spreading along with polymer chains rather than concentrating, which prevents the structural failure and endows SLA-Silivoxels with super-elastic properties. As evidenced by the finite element analysis (FEA, Fig. 2b) of the elastic response, when the lattice model is subjected to certain compressive displacement, no stress concentration is observed on the structure surface while the strain distribution is uniform across the cross-sectional area. The TPMS structure could have a large mechanical recovery capability with a high loading of silivoxels. The synergistic effect from hydrogel molecular chains and porous silivoxels affects the recoverable strain, which results from the applied compression stress evenly distributed on the surfaces avoiding the stress concentration.

The SLA-silivoxels after drying maintain the shape and dimension, which resist the shrinkage as compared with the SLA samples without silivoxels (Fig. 2c). The SEM images show the wrinkle morphology for the SLA sample without silivoxels while the existence of silivoxels resists surface wrinkle (Fig. 2d, e). With the concentration of silivoxels < 28.5 wt%, the maximum recovery strain is 80% while silivoxels concentration increases from 28.5 wt% to 75 wt%, the maximum recovery strain maintains at 75% (Fig. 2f and Supplementary Figs. 8–10). The elastic strength *Y*, and Young’s modulus *E* for the samples with different silivoxel concentrations are shown in Supplementary Fig. 10. For the samples without hierarchical porous silivoxels, the *Y* and *E* are 0.0716 MPa and 0.0895 MPa, respectively, and with increasing silivoxel concentration to 28.5 wt%, the *Y* and *E* increase ~12×, to 0.8153 MPa and 1.1 MPa, respectively. Figure 2f compares the temperature dependence of thermal conductivity of SLA-Silivoxel from 30 °C to 106 °C. The SLA sample without porous silivoxels shows high thermal conductivity of 0.21 W m^−1^ K^−1^ at 30 °C, and with temperature increasing, the thermal conductivity increases gradually to 0.225 W m^−1^ K^−1^. Due to the low thermal conductivity of porous silivoxels, thermal conductivity of the SLA-Silivoxels decreases to 0.06 mW m^−1^ K^−1^ when the concentration of silica reaches 75 wt%, compared with that (0.21 W m^−1^ K^−1^) of SLA sample without silivoxels. Figure 2g compares thermal conductivity and Young’s modulus, strength of different hybrid material systems, confirming mechanical robustness and low thermal conductivity of SLA-Silivoxles^15–17^. The hybrid materials generally show high thermal conductivity of 0.1–0.6 W m^−1^ K^−1^, in contrast, thermal conductivity of SLA-silivoxels could achieve at 0.06 W m^−1^ K^−1^. The enhanced thermal insulation performance results from high loading of silivoxels (75 wt%) in the flexible hybrid networks, while silivoxels also dictate thermal insulation performance compared with the limited loading of hollow nanoparticles (<30 v%) in the polymer matrix shown in Fig. 2g. SLA-silivoxels can find promising thermal management applications on flexible electronics and wearable devices.

**Fig. 3:**
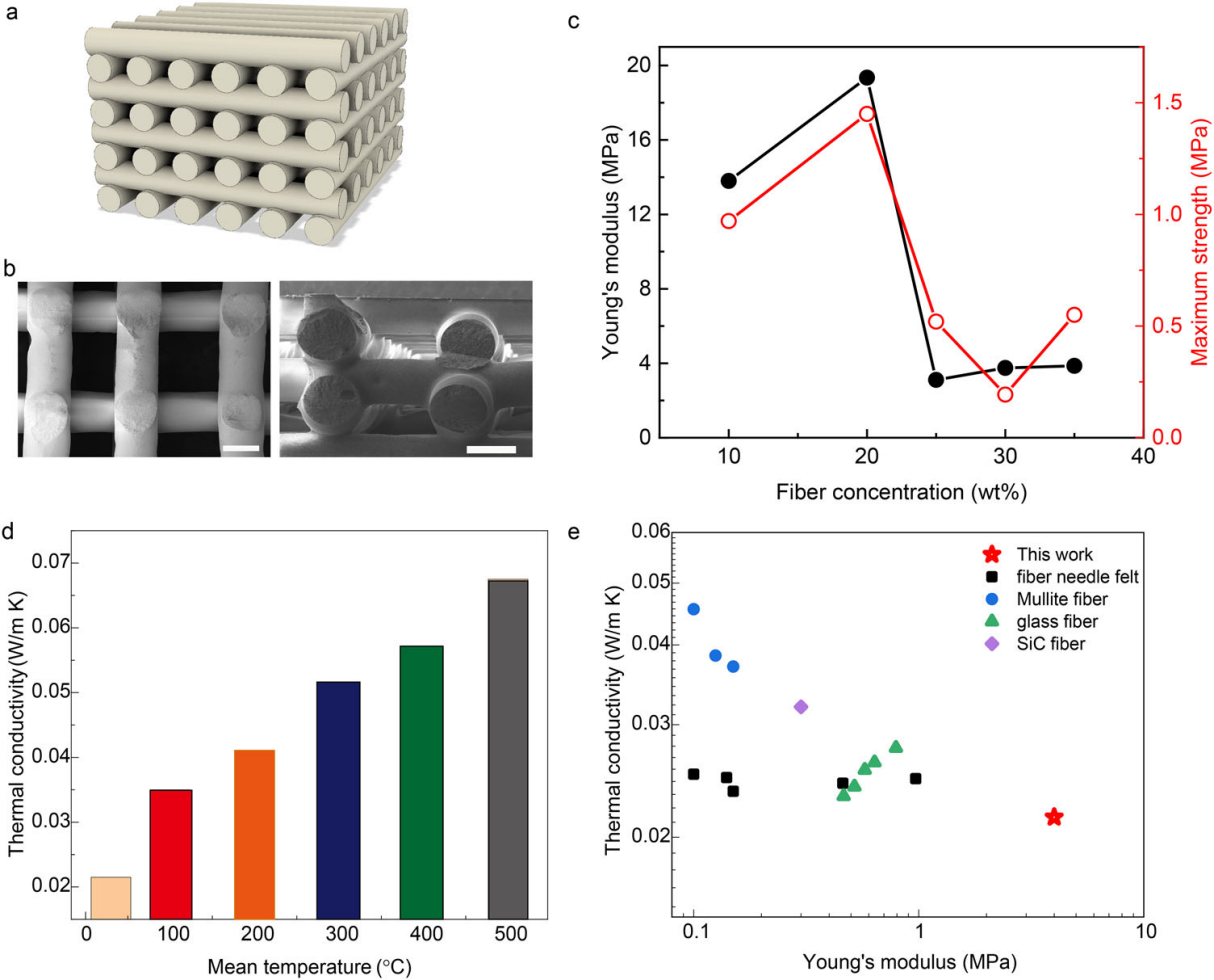
Characteristics of DIW-silivoxels. **a** The CAD model of the hollow pile-up structure. **b** SEM images of the hollow wood-pile structure of DIW-silivoxels. The scale bars are 400 μm. **c** Young’s modulus and maximum strength vs. fiber concentration. **d** Thermal conductivity vs. temperature of DIW-silivoxels. **e** Comparison of thermal conductivity vs. Young’s modulus of literatures and this work.

Mechanical reinforcement of porous silivoxels through the fiber additives could be further achieved by the direct ink writing (DIW-Silivoxels), on which an example pile-up structure demonstrates high-resolution capability (Fig. 3a, b and Supplementary Fig. 11). The mechanical properties of DIW-Silivoxels are evaluated through the uniaxial compression tests and multiple-cycles stress-strain curves (Supplementary Fig. 12). After the yield stress is reached, the printed porous structure can be densified by increasing the compression strain to increase the compression strength. For the 2nd compression cycle with the strain reaching 4%, the maximum strength of DIW-Silivoxels could reach 1.5 MPa. The load drop during the peak load indicates the failure of DIW-Silivoxels during the compression condition. With the fiber concentration higher than 20 wt%, the DIW-Silivoxels become soft, and the maximum load curves drop compared with the samples with 10 wt% and 20 wt% fibers (Supplementary Fig. 12). A higher volume of fibers in DIW-Silivoxels can trigger the delamination during the compression. With the fiber concentration increasing from 10 wt% to 30 wt%, the porosity drops from 82% to 70% (Supplementary Fig. 12). Figure 3c shows the Young’s modulus and strength of DIW-Silivoxels, with the optimum Young’s modulus of 20 MPa and strength of 1.5 MPa for 20 wt% fiber additives. Beyond 20 wt%, the DIW-Silivoxel becomes compliant and soft with a constant Young’s modulus of 4 MPa and strength of 0.5 MPa. The temperature dependence of thermal conductivity of DIW-Silivoxel in Fig. 3d shows the increase of thermal conductivity from 0.035 W/m K to 0.07 W/m K with the temperature increase from 100 °C to 500 °C. Figure 3e compares the thermal conductivity vs. Young’s modulus of DIW-Silivoxel and fiber reinforced hybrid materials^18,19^, where DIW-Silivoxel shows a lower thermal conductivity and higher compression rigidity. The interactions between porous silivoxel assemblies and fiber reinforcement benefit the enhanced mechanical compression rigidity while the synergistic contribution of hierarchical porous hybrid networks exhibit high thermal insulation (Fig. 3e). The printed silivoxels with high shape fidelity and complex constructs exhibit high mechanical tunability and lightweight, which promise it for thermal management applications.

**Fig. 4:**
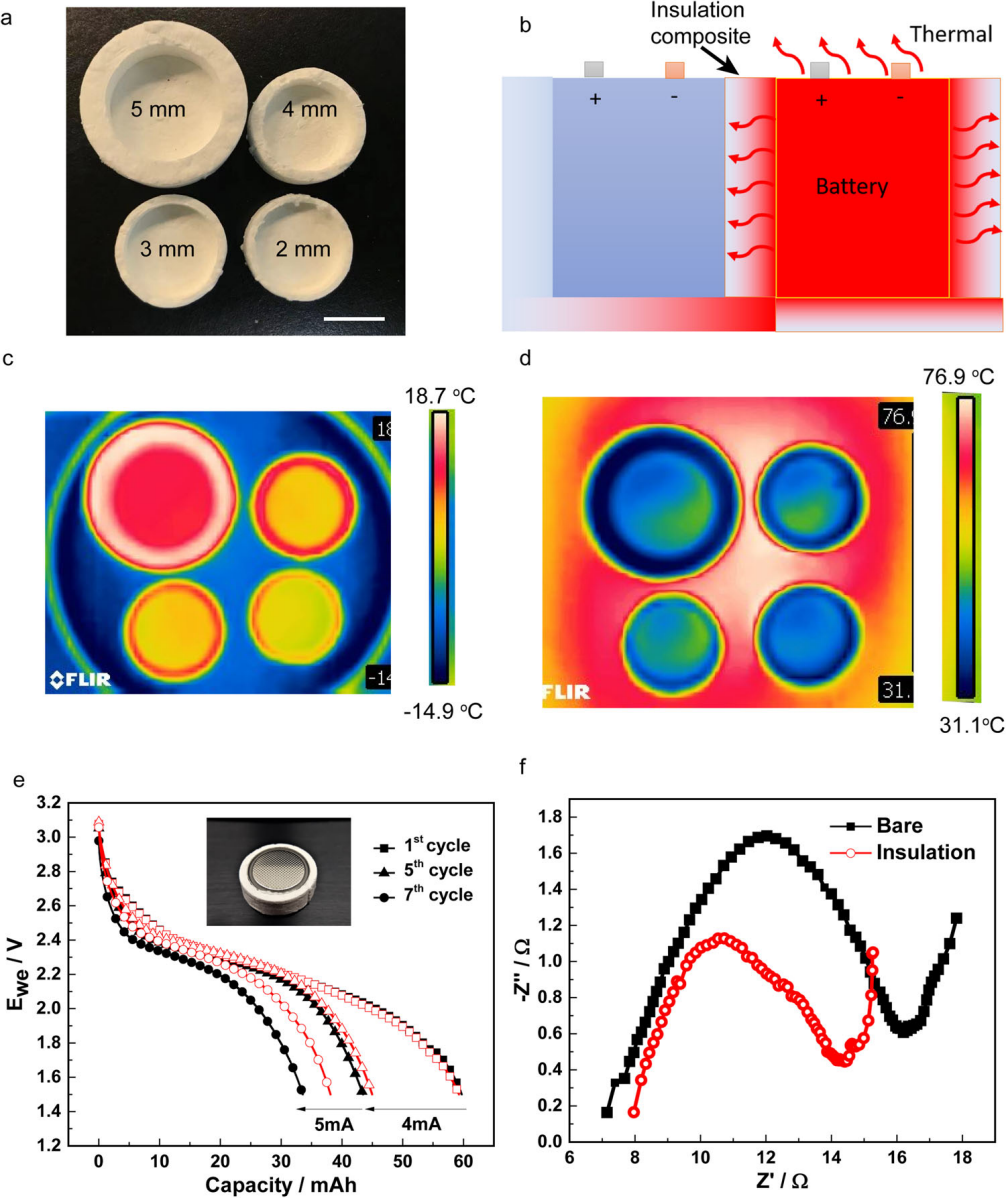
Additive manufacturing silivoxels for battery thermal management. **a** The DIW-silivoxels battery cases with thickness of 2 mm, 3 mm, 4 mm, and 5 mm. Scale bar is 10 mm. **b** Thermal mitigation scheme of battery packs. **c**, **d** IR image of the DIW-silivoxels cases with different thicknesses on a cold surface (−14.9 °C) and a hot surface (76.9 °C). **e** Charging/Discharging profiles of lithium-ion batteries with (open symbol) and without (closed symbol) DIW-silivoxel cases for thermal protection on a hotplate at 70 °C. **f** Nyquist plots of lithium-ion batteries with and without DIW-silivoxel cases for thermal protection on a hotplate at 70 °C.

Thermal insulation materials protect lithium-ion rechargeable battery from the spread of overheating. The local thermal runaway or burning accidents induced high-temperature hotspots affect the battery assemblies to cause the local damage, and such thermal runaway mitigation strategies are necessary for thermal management systems, such as wearable devices, micro-electromechanical systems, smartphones, and electric automobiles^20,21^. A variety of DIW-silivoxel battery cases for lithium-ion battery cells are thus printed with the different thicknesses of 2 mm, 3 mm, 4 mm, and 5 mm (Fig. 4a) to evaluate its battery protection from the extreme environment and thermal runaway. Figure 4b shows the scheme of thermal migration of a pack of rechargeable batteries under the protection of the DIW-silivoxel battery cases. Thermal infrared (IR) images of the DIW-Silivoxel battery cases with different thicknesses reveal a thermal resistance on the cold (−14.9 ^o^C) and hot (76.9 °C) environment, as shown in Fig. 4b-c. The delta-temperature between the top and bottom surface of the DIW-Silivoxel displays the increasing value from 30 to 45 °C for the samples with the thickness from 2 mm to 5 mm, promising thermal runaway mitigation for lithium-ion battery applications. To illustrate the utility of DIW-silivoxels in thermal protection of batteries, we further evaluate the rechargeable batteries heated at 70 ˚C with/without the DIW-silivoxels battery cases for thermal protection, and the resulting galvanostatic charging/discharging profiles are shown in Fig. 4e. The initial discharge capacities of both cells are ~60 mAh, which are close to its nominal discharge capacity (65 mAh). At a discharge current of 4 mA, both of the cells showed significant capacity loss, which is 18% for the one with insulation and 27 % for the one without insulation at the fifth charging/discharging cycle. At a higher discharge current (5 mA), the capacity loss is more severe, and the values increased to 36% and 44% for the ones with and without insulation, respectively, at the 7th charging/discharging cycle. The much lower capacity loss observed with the insulation layer demonstrates the great potential of the DIW-silivoxels in mitigating the thermal aging effect of batteries. To identify the origin of the smaller capacity loss associated with the DIW-silivoxels insulation case, electrochemical impedance spectroscopy (EIS) is employed to investigate the dynamic behavior of the cells after heating. As shown in the Nyquist plot in Fig. 4f, there are a depressed semicircle in the high-frequency range and a tail in the low-frequency range. Compared to the negligible difference of series resistance (*R*_s_, the first point when *Z*” = 0), a smaller charge transfer resistance (*R*_ct_, the semicircle diameter) and a steeper slope of the tail are observed in the cell with the DIW-silivoxel insulation case protection, suggesting a smaller resistance of charge transfer at the electrode/electrolyte interface and a fast ion diffusion process, respectively^22^. The result demonstrates that the insulation case can ensure facile charge/discharge kinetics via minimizing the change of charge transfer resistance and maintaining a fast lithium-ion diffusion rate when operating at high temperature. In addition to thermal runaway mitigation, the DIW-silivoxels also exhibit an excellent soundproof performance (Supplementary Fig. 13), where the sound intensities decrease by 28%, 28% and 32% under 500 Hz, 800 Hz and 1200 Hz, respectively compared with the background noise. The soundproof efficiency of DIW-silivoxels is 2–3 times higher than that of polystyrene foam from 500 Hz to 800 Hz while the soundproof efficiency of DIW-silivoxels is 7 times higher than that of polystyrene foam under the frequency of 1200 Hz.

In conclusion, we present an agile additive manufacturing of architectured porous silivoxels. The tunable mechanical robustness can be achieved through the orthogonal surface hybridization of silivoxels, which can be 3D-printed using stereolithography and direct ink writing. The convergence of complex and optimized design (e.g., TPMS structure), additive and digital fabrication (e.g., SLA and DIW), efficient and programmable material synthesis (e.g., silivoxels) offered a unique solution to spatially assemble the microscopic building blocks into a tunable and controllable macroscopic structure with desired thermal insulation and mechanical properties, retaining low thermal conductivity of 19.1 mW m^−1^ K^−1^, flexible compressive recovery strain (85%), and tailored mechanical strength from 0.2 MPa to 1.4 MPa. In addition, the lightweight 3D-printed silica parts promise thermal aging mitigation for energy-critical battery applications.

## Methods

### Materials and printing procedure

#### Porous silivoxels synthesis

The porous silivoxels are prepared by dissolving 18.75 g of Cetyl Trimethyl Ammonium Bromide (CTAB, Sigma-Aldrich), 15 mL mesitylene, and 1.5 mL ammonia in 1500 mL deionized water under various temperatures (0, 30, 60, 100°C). The solution is mixed for 4 hours using a magnetic stirrer (Thermo Scientific) operating at 800 rpm. Then, 15 mL Tetraethyl orthosilicate (TEOS, Alfa Aesar) is slowly added into the solution. Keep the solution in the oven under 60 °C for evaporation. The dried porous silivoxel particles are heat-treated under 480 °C for 1 h to eliminate the organic residues. A ball-milling machine is used to grind the silivoxel particles for subsequent 3D printing.

#### Mask image projection based stereolithographic 3D printing

The printable precursor is prepared by mixing a 15 v% of Poly (ethylene glycol) diacrylate (PEGDA, Mn700, Sigma-Aldrich), 0.5 wt% photo-initiator (Lithium phenyl-2,4,6-trimethylbenzoylphosphinate, purity ≥ 95%, Sigma-Aldrich), 0.05 wt% photo-absorber (Quinoline yellow, purity≥ 95%, Sigma-Aldrich), and ball-milled porous silivoxels. The photo-initiator and photo-absorber are used as purchased. Then a custom-built SLA printer with a bottom-up configuration is used to print the high-precise architectures. The light source of the projector is 385 nm ultraviolet (UV) light, with a 1080 P projector (Wintech PRO4500, Texas Instrument). The Eiffel tower CAD (Computer-aided design) model is sliced to mask images using a custom-programmed slicing software with a layer-thickness of 50 µm. A custom-programmed control software synchronizes the mask image projection and the build-platform motion. The printed parts are dried in a humidity-controlled environment at 80 °C for 12 h.

#### Direct ink writing process

The printable colloidal ink is prepared by mixing the sintered porous silivoxels in deionized water at 70 wt%. The aluminoborosilicate fiber (Unifrax) is added to the colloidal ink by 30 wt% of the porous silivoxels. The dispersing agent (Darvan 811, Vanderbilt Minerals) is added by 2 wt% of deionized water to disperse the silivoxel particles. Viscosity modifier (HPMC, Hydroxypropyl methyl cellulose, H7509, Sigma-Aldrich) is added to the ink to at a concentration of 1.1 wt% of the deionized water to adjust the rheology property^23^. The ink is mixed homogeneously for 12 hours on a magnetism stirrer. The ready-to-print colloidal ink exhibits a shear-thinning rheological behavior. A customized extrusion-based 3D printer is used for the direct writing of the porous silivoxels ink. An air-compressor is used to provide pneumatic pressure (3.2 psi) for extrusion process. The extruder consists of a helix-locked 400-µm nozzle (Nordson EFD). The extruder path is generated by the Slic3r software (slic3r.org), the infill pattern is set as rectilinear, and the layer thickness is 250 μm for printing the high-aspect ratio vase model (Fig. 1c).

#### Microstructural analysis

The microstructure of silivoxels and printed samples are imaged using Carl Zeiss AURIGA scanning electron microscopy (SEM) at an accelerating voltage of 3 kV. Nominally a thin layer of Au is sputtered to avoid charging effect. TEM images of samples are recorded on a JEOL-2100 high-resolution transmission electron microscopy (TEM) at 200 kV. BET analysis is performed on a Tristar II 3020 (Micromeritics Corp. Atlanta, GA). The specific surface area (SSA) and the pore size distributions are evaluated with low-temperature (77 K) nitrogen (N_2_) adsorption–desorption isotherm measurement method. The pure nanoparticles are degassed at 300 °C for 1 h before analysis. The surface areas are calculated with Brunauer–Emmett–Teller (BET) theory using isotherm adsorption data at P/P_0_ from 0.05 to 0.30. Fourier Transform Infrared (FTIR) Spectra are acquired in attenuated total reflection mode (ATR-FTIR spectroscopy) with a Bruker VERTEX 70 on ZnSe substrate, and atmospheric compensation is implemented during the measurement.

#### Thermal measurement

The home customized thermal conductivity measurement follows the ASTM C518 standard thermal conductivity procedure. The flux sensor from Fluxteq company is calibrated with the reference commercial extruded polystyrene thermal insulation material. Temperature dependence thermal conductivity measurement is performed on TPS 2200. The thermal insulation performance is evaluated using an infrared camera (Fotric 225 Pro Thermal Camera). Electrochemical measurements of the lithium manganese dioxide rechargeable batteries (model Maxell ML2032) are performed by a VMP3 instrument (BioLogic Science Instruments). The cells are charged/discharged galvanostatically between 1.5 and 3.3 V at 70 °C. To achieve a good comparison, the intentionally higher discharged currents (4 and 5 mA) are used compared to the nominal discharge current (0.2 mA). The AC impedance spectra are collected by applying a sine wave with an amplitude of 5 mV over a frequency range of 1 kHz to 100 mHz.

#### Mechanical testing and finite element analysis

The mechanical properties of composite nanoparticles are studied using an MTS universal testing machine with an ultra-sensitive load cell. The sample sizes are 1-inch cubes. The investigation of the finite element model is performed in Abaqus solver from Dassault Systems. All degrees of freedom of the bottom plate are fixed. Ten-node tetrahedral element is used for meshing the Schwarz primitive model. The mesh convergence analysis is also performed.

## Supplementary information

Supplementary Information

## Data Availability

The data supporting the findings of this study are included within this article and its supplementary files. Any additional information is available from the authors upon request.
