# Supplementary material for: Tailoring thermal insulation architectures from additive manufacturing
Source: Nat Commun. 2022 Jul 25;13:4309. doi: 10.1038/s41467-022-32027-3 (PMC9314391; doi:10.1038/s41467-022-32027-3)
Supplement: Supplementary file 1 — Supplementary Information [file 41467_2022_32027_MOESM1_ESM.pdf]

Supplementary information

Tailoring thermal insulation architectures from additive manufacturing

Lu An[1,⊥], Zipeng Guo[2,⊥], Zheng Li[1,⊥], Yu Fu[3], Yong Hu[1], Yulong Huang[1], Fei Yao[3,*], Chi Zhou[2,*], Shenqiang Ren[1,4,5,*]

[1] Department of Mechanical and Aerospace Engineering, University at Buffalo, The State University of New York, Buffalo, NY 14260, USA

[2] Department of Industrial and Systems Engineering, University at Buffalo, The State University of New York, Buffalo, NY 14260, USA

[3] Department of Materials Design and Innovation, University at Buffalo, The State University of New York, Buffalo, NY 14260, USA

[4] Department of Chemistry, University at Buffalo, The State University of New York, Buffalo, NY 14260, USA

[5] Research and Education in Energy, Environment & Water (RENEW) Institute, University at Buffalo, The State University of New York, Buffalo, NY 14260, USA

[⊥] Equal Contributions

[*] Corresponding Authors: feiyao@buffalo.edu; chizhou@buffalo.edu; shenren@buffalo.edu

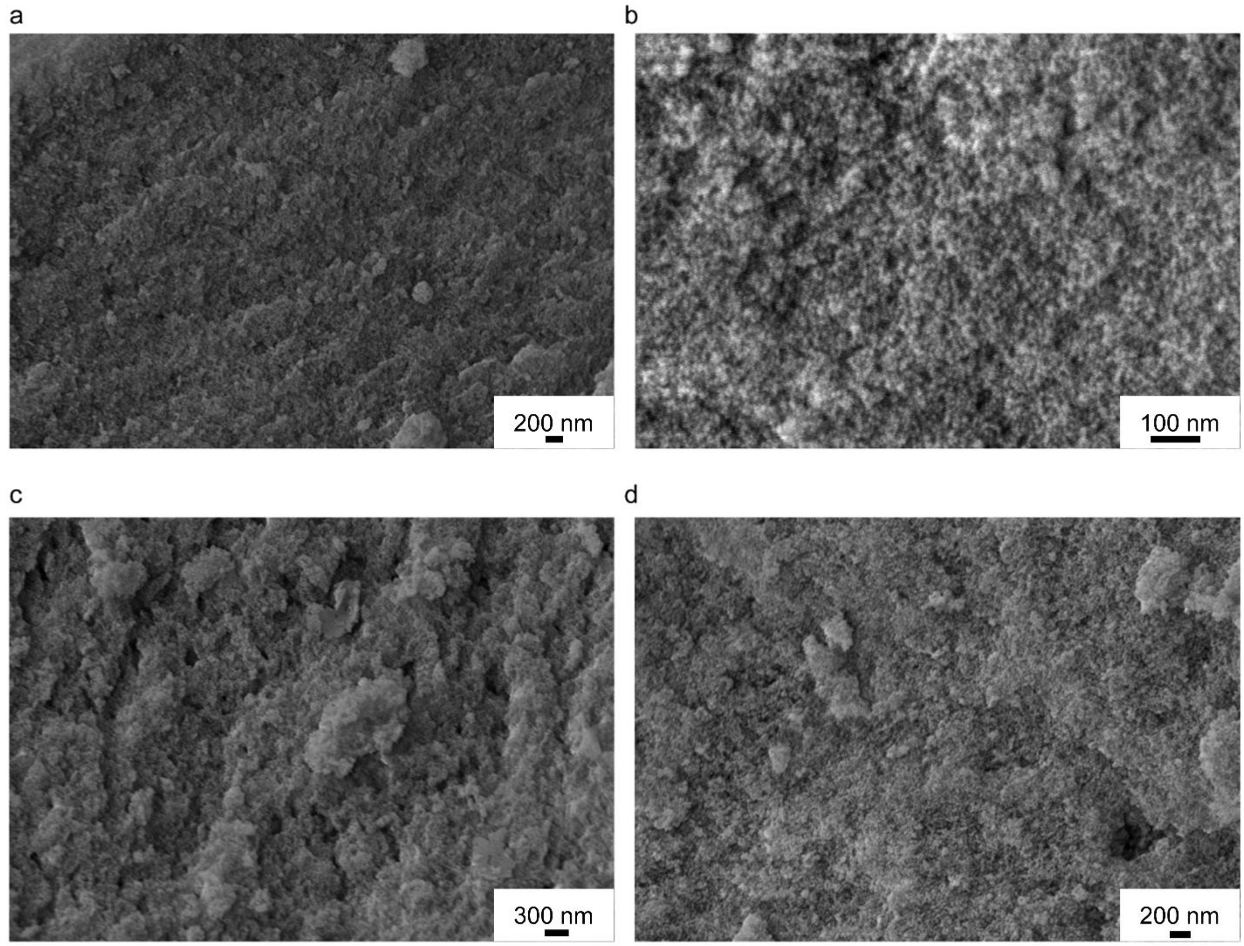

Supplementary Figure 1. SEM (Scanning electron microscope) images of silivoxels with different reaction temperatures. (a) Reaction temperature of 0℃, (b) 30℃, (c) 60℃, and (d) 100℃, after sintering treatment under 480℃ for 1h.

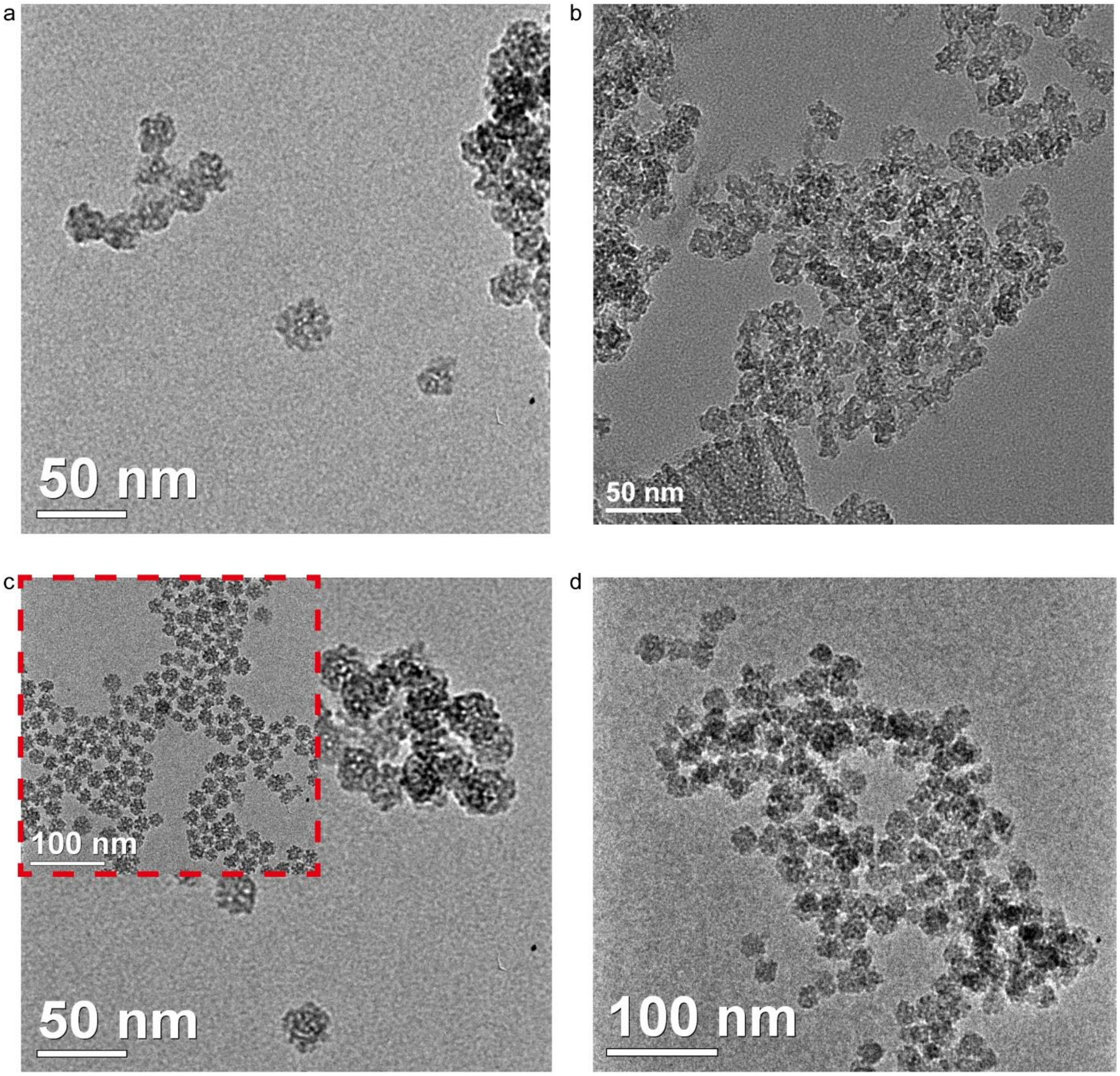

Supplementary Figure 2. TEM (Transmission electron microscopy) images of silivoxels with different reaction temperatures. (a) Reaction temperature of 0℃, (b) 30℃, (c) 60℃, and (d) 100℃, after sintering treatment under 480℃ for 1h.

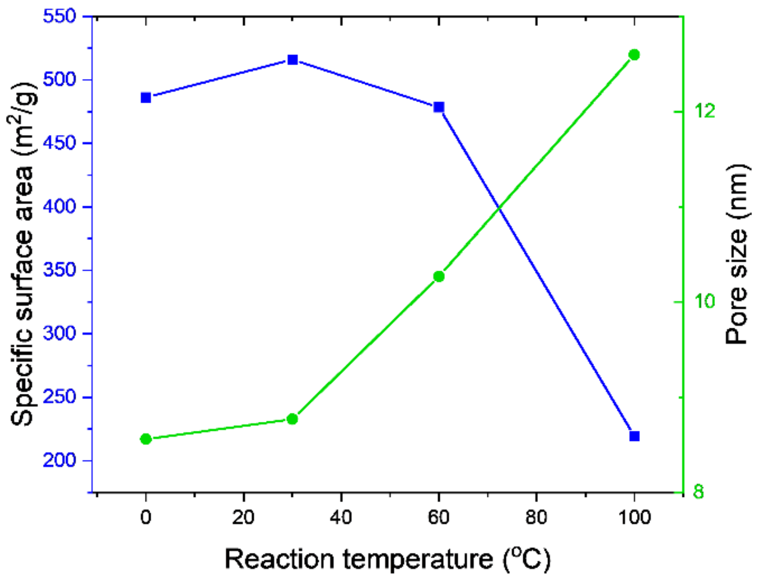

Supplementary Figure 3. Specific surface area (SSA) and pore size characterizations of silivoxels. Blue curve represents SSA and green curve represents pore size.

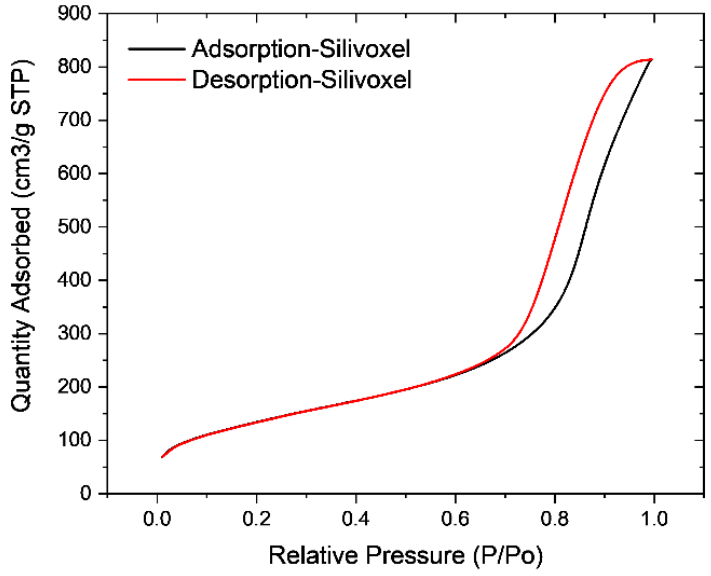

Supplementary Figure 4. Adsorption and desorption isotherm curves of silivoxels. Nitrogen is used for the adsorption-desorption analysis.

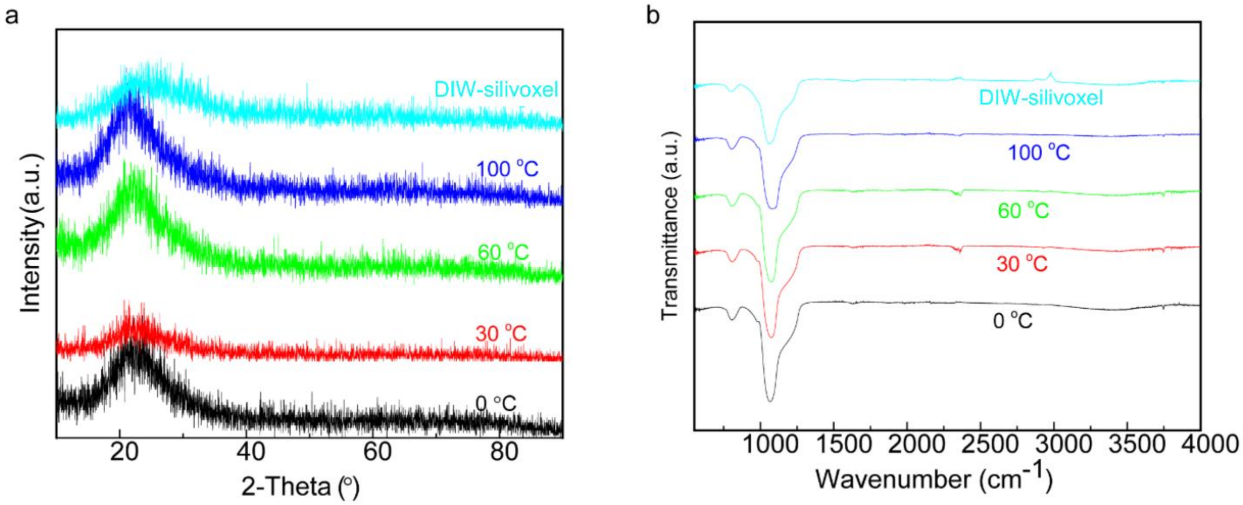

Supplementary Figure 5. Structural analysis of silivoxels. (a) XRD (X-ray diffraction) spectra and (b) FTIR (Fourier-transform infrared) spectra of silivoxels with different reaction temperatures and DIW-silivoxel after sintering treatment under 480℃ for 1h.

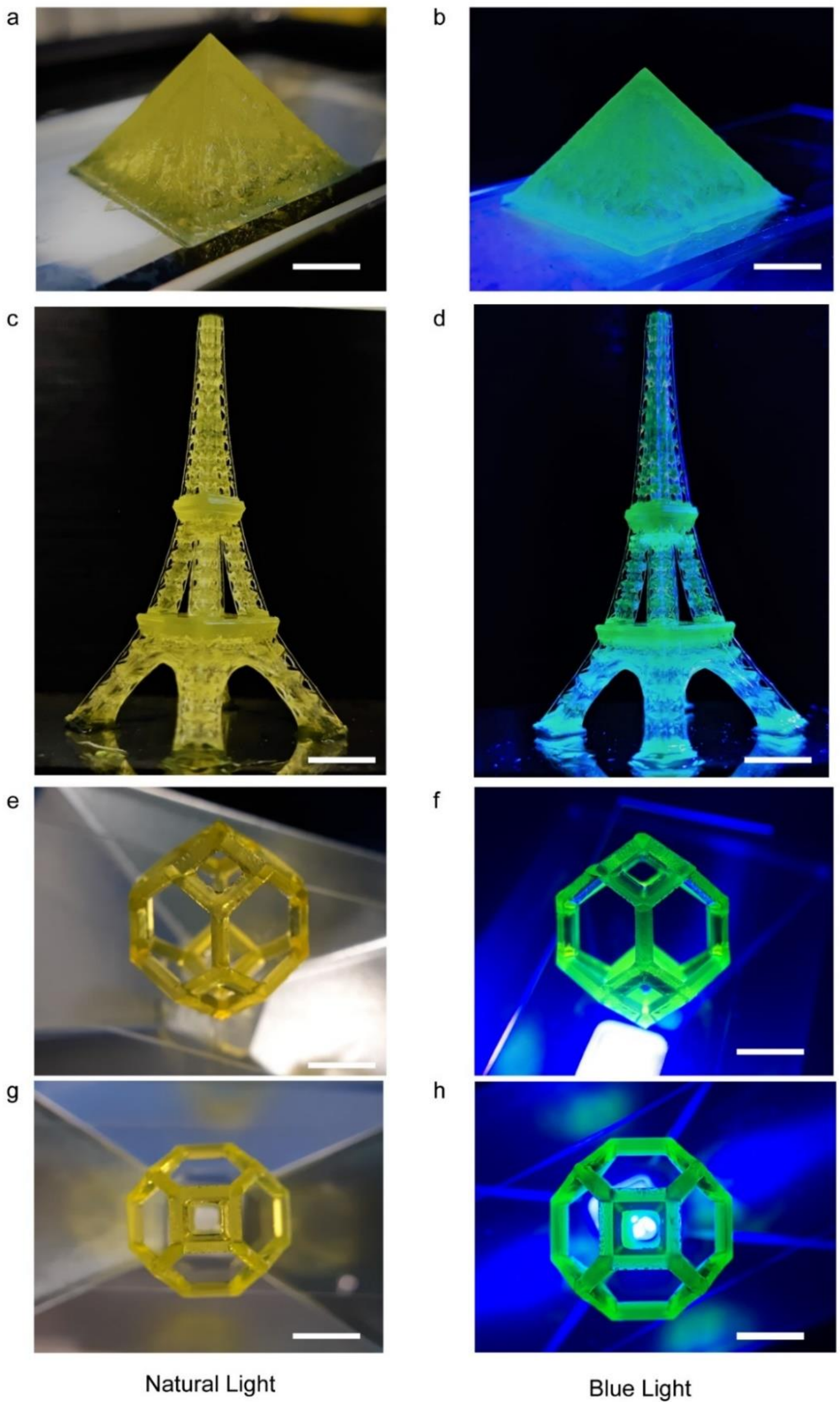

Supplementary Figure 6. The miniaturized architectures of SLA-silivoxels under natural light and blue light sources. The scale bars are 2 cm. (a) and (b) are hollow pyramid model. (c) and (d) are Eiffel tower model. (e)-(h) are hollow cage model.

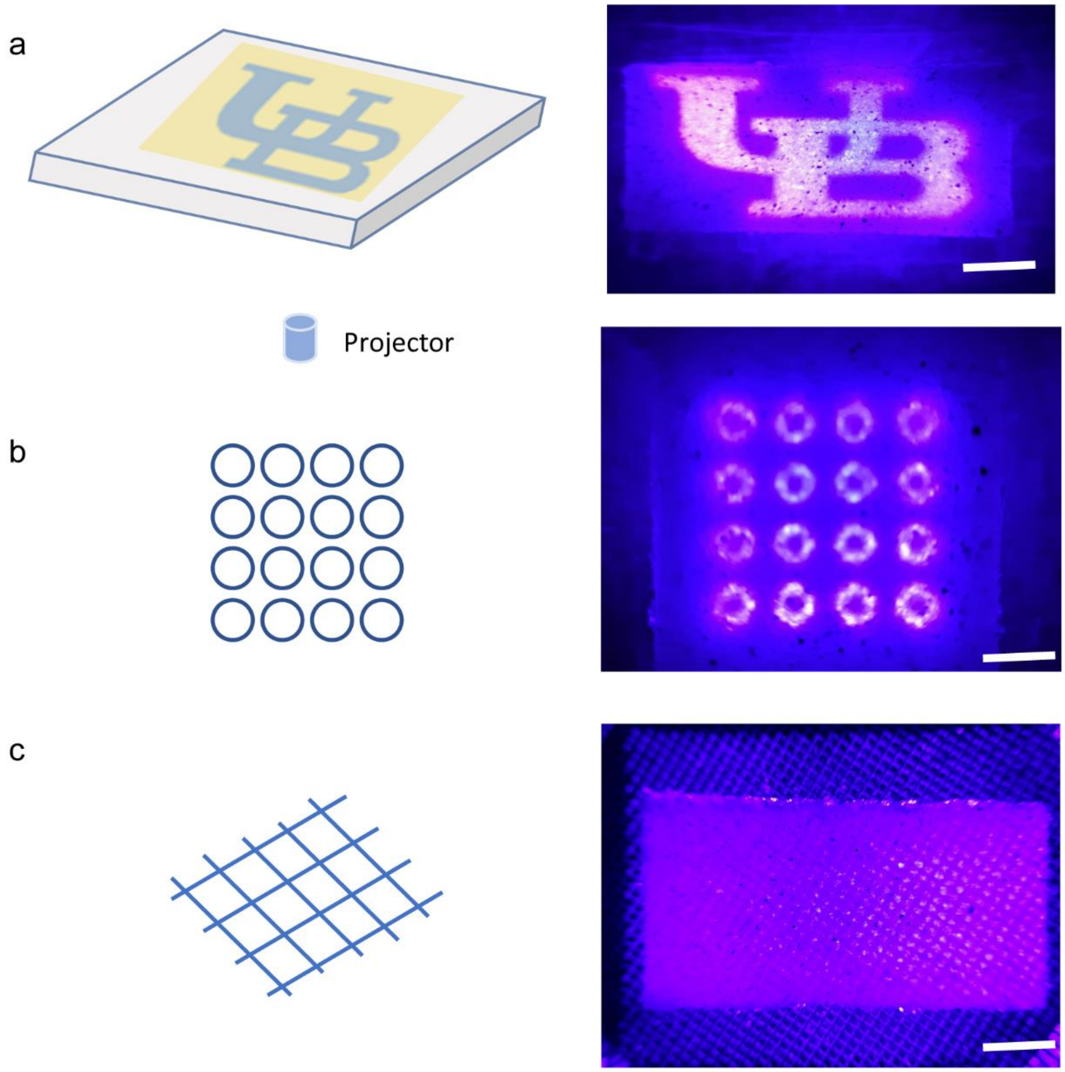

Supplementary Figure 7. Demonstration of optical transmission of SLA-silivoxels under blue light. The scale bars are 5 mm. (a) Scheme of the optical transmission demonstration with a mask image of UB logo. (b) Mask image of a circle matrix. (c) Mask image of a mesh grid.

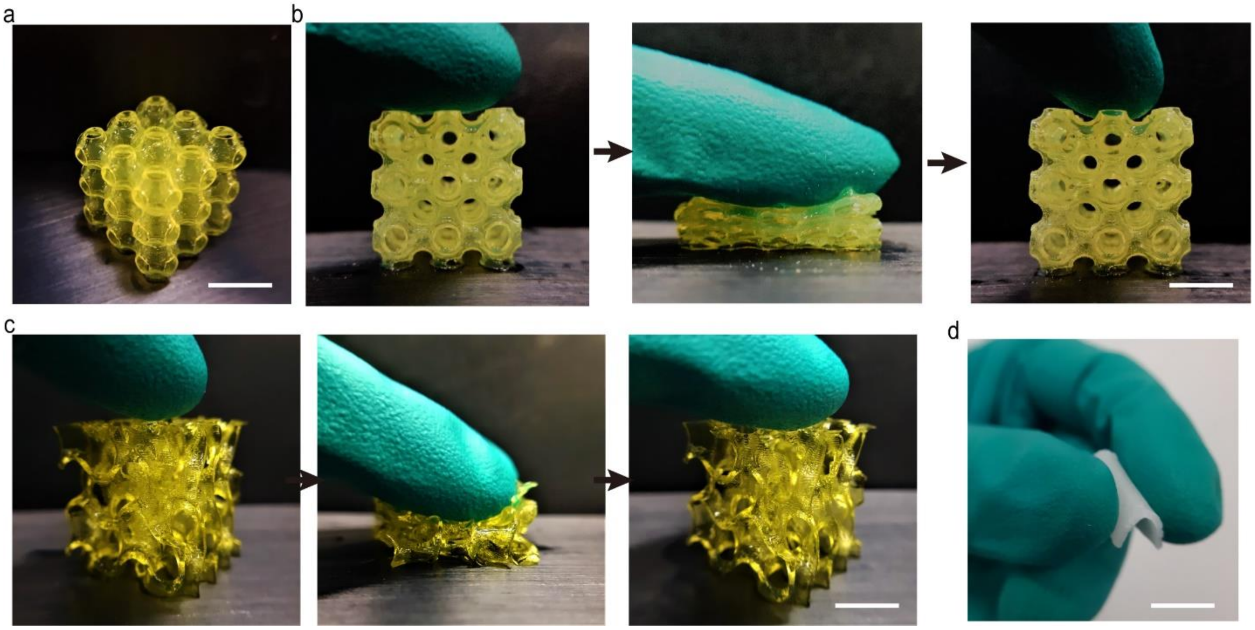

Supplementary Figure 8. Demonstration of mechanical properties of SLA-silivoxels. (a) Optical image of SLA-silivoxels (Triply periodic minimal structure-primitive model, TPMS-P model). (b) Compression recovery of lattice (TPMS-P model). (c) Compression recovery of lattice (TPMS-Gyroid model). (d) Bendability of SLA-silivoxels. The scale bars are 5mm.

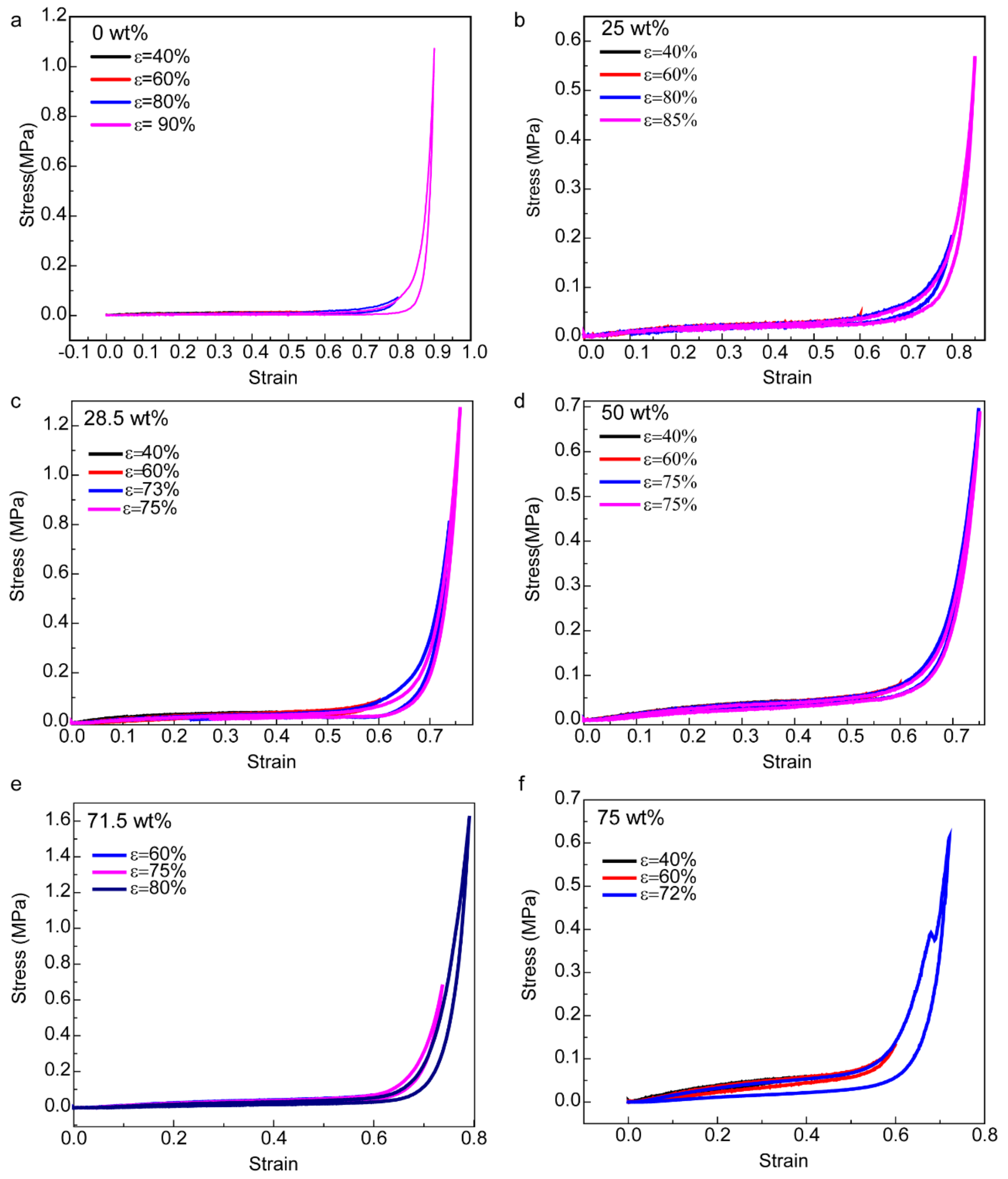

Supplementary Figure 9. Multiple compression stress-strain curves of SLA-Silivoxel lattice with silivoxel concentration of (a) 0 wt%, (b) 25 wt%, (c) 28.5 wt%, (d) 50 wt%, (e) 71.5wt%, and (f) 75 wt%.

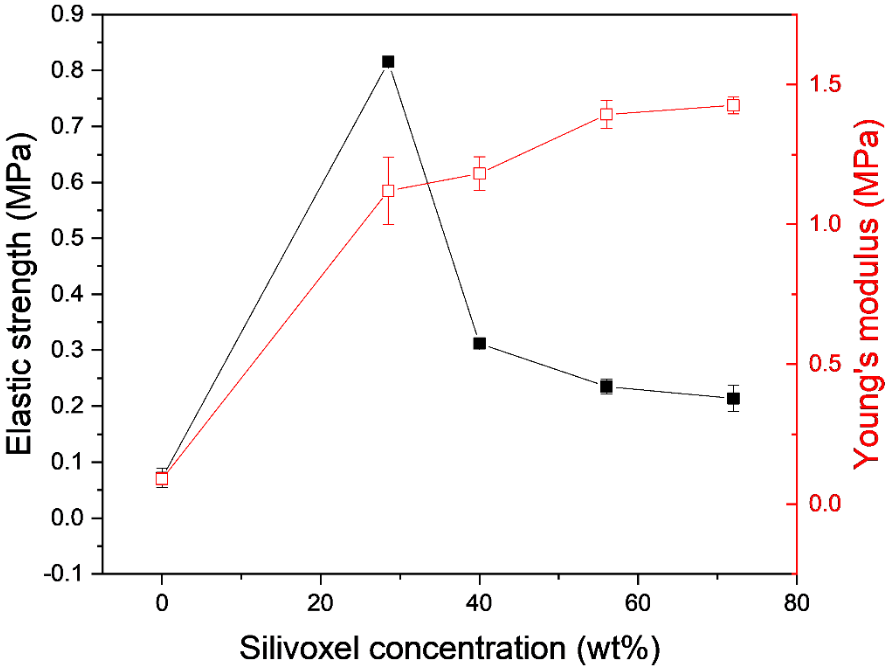

Supplementary Figure 10. Mechanical characteristics of SLA-silivoxels. Black curve represents elastic strength and red curve represents Young’s moduls.

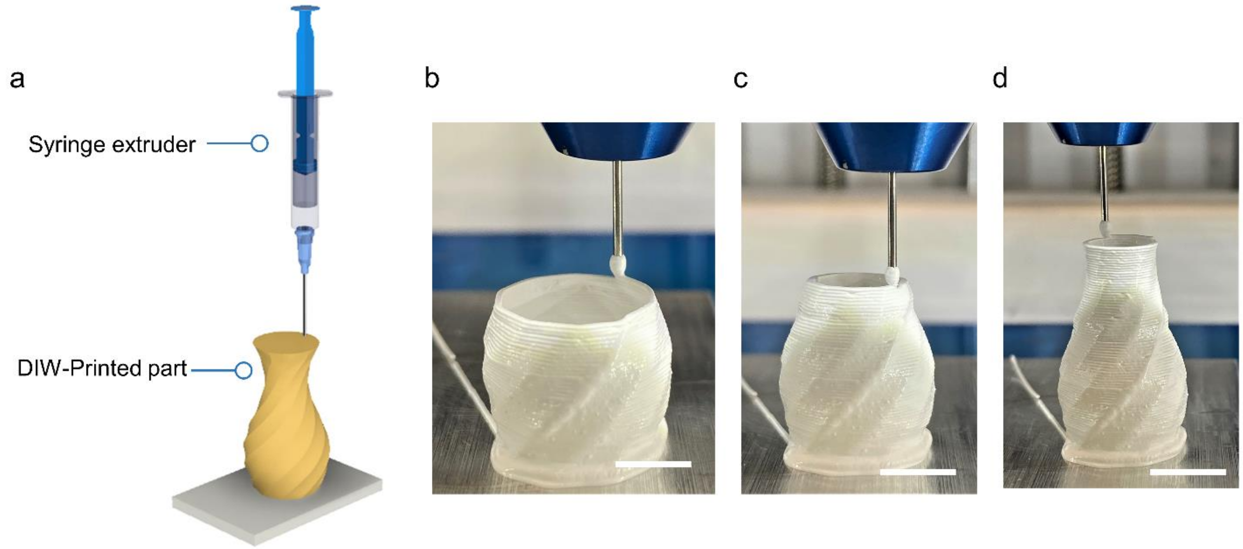

Supplementary Figure 11. Direct ink writing process of silivoxel ink in vase structure. (a) Schematic diagram of direct ink writing. (b)-(d) Sequential photographs of DIW process. The scale bars are 5mm.

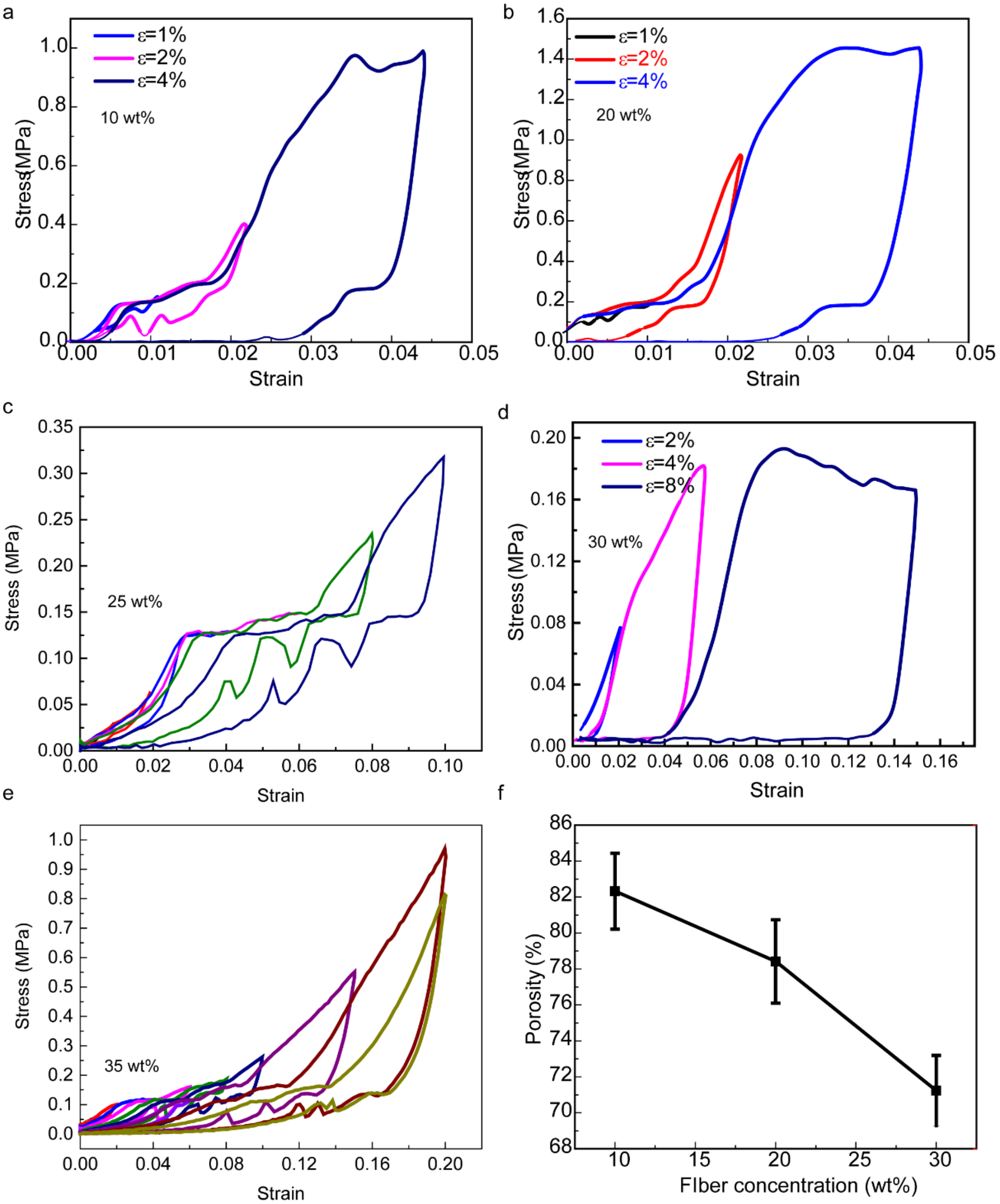

Supplementary Figure 12. Uniaxial compression of DIW-Silivoxels with fiber incorporation of (a) 10 wt%, (b) 20 wt%, (c) 25 wt%, (d) 30 wt%, (e) 35 wt%, and (f) Porosity vs. fiber concentration.

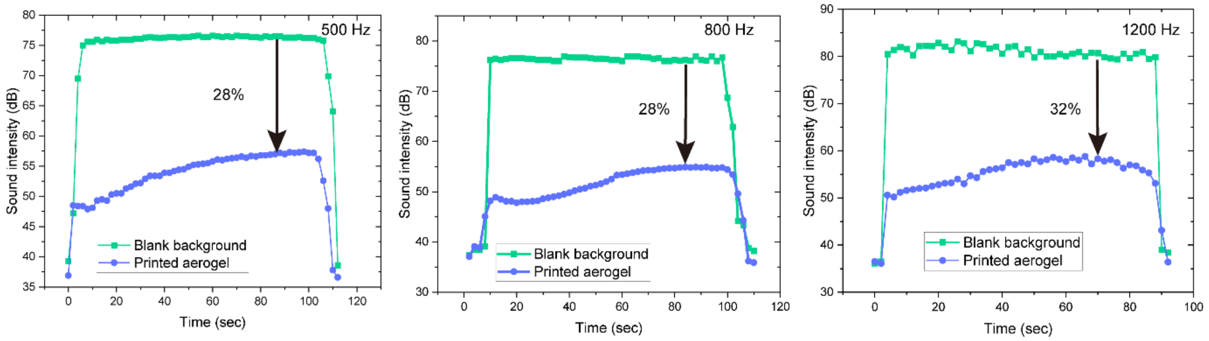

Supplementary Figure 13. Soundproof performance denmonstration of DIW-Silivoxels samples. The tests are performed under frequencies of 500 Hz, 800 Hz, and 1200 Hz.
